# Multisensory System for Long-Term Activity Monitoring to Facilitate Aging-in-Place

**DOI:** 10.3390/s23208646

**Published:** 2023-10-23

**Authors:** Sergio Lluva-Plaza, Ana Jiménez-Martín, David Gualda-Gómez, José Manuel Villadangos-Carrizo, Juan Jesús García-Domínguez

**Affiliations:** 1 Department of Electronics, University of Alcalá, 28801 Madrid, Spain; sergio.lluva@uah.es (S.L.-P.); ana.jimenez@uah.es (A.J.-M.); jm.villadangos@uah.es (J.M.V.-C.); 2 Department of Signal Theory and Communications, University Rey Juan Carlos, 28943 Fuenlabrada, Spain; david.gualda@urjc.es

**Keywords:** healthy ageing, aging in place, activity monitoring, frailty assessment, routine detection

## Abstract

Demographic changes and an ageing population require more effective methods to confront the increased prevalence of chronic diseases which generate dependence in older adults as well as an important rise in social expenditure. The challenge is not only to increase life expectancy, but also to ensure that the older adults can fully enjoy that moment in their lives, living where they wish to (private home, nursing home, …). Physical activity (PA) is a representative parameter of a person’s state of health, especially when we are getting older, because it plays an important role in the prevention of diseases, and that is the reason why it is promoted in older adults. One of the goals of this work is to assess the feasibility of objectively measuring the PA levels of older adults wherever they live. In addition, this work proposes long-term monitoring that helps to gather daily activity patterns. We fuse inertial measurements with other technologies (WiFi- and ultrasonic-based location) in order to provide not only PA, but also information about the place where the activities are carried out, including both room-level location and precise positioning (depending on the technology used). With this information, we would be able to generate information about the person’s daily routines which can be very useful for the early detection of physical or cognitive impairment.

## 1. Introduction

There has been a worldwide rise in the population of older adults and with it the number of people with chronic diseases and dependence has also risen, representing a significant increase in social and health care costs [1]. On the other hand, habits have changed, and older adults have expressed their desire to age in place [2]. This term, aging-in-place, has been defined as: “the ability to live in one’s own home and community safely, independently, and comfortably, regardless of age, income, or ability level” [3]. However, without the appropriate support they may suffer an increased risk of accidents and injuries as well as comorbidity. IoT-based tools, such as environmental sensing platforms, can help the older adults to continue in their own homes, providing environments that enhance a healthy, safe, and independent living [4,5].

Physical function variation (aerobic capacity, gait speed and muscle strength) has been proposed as a biomarker of healthy aging because of its direct relationship with adverse health events [6]. Physical Activity (PA) plays an important role in disease prevention; tracking this in older adults and monitoring their behavioural patterns provide relevant information about their functional, cognitive and social health status. It provides important information which furthers an understanding of the person’s ability to maintain their functionality and independence, and that is why it is essential to promote PA in older adults [7,8]. Some authors point out that reduced levels of PA and mobility are important predictors of frailty and a reduced quality of life [9,10,11,12].

Daily physical activity (DPA) estimation is a challenging task because of its multi-dimensional nature. PA is defined as any bodily movement, encompassing exercise, sports and other activities performed as part of daily living [7]. Therefore, an objective quantification can be carried out through many different parameters; for example, step counting, intensity and type of DPA (eating, sleeping, etc.) or through spatiotemporal measures of gait like stride length, gait velocity or gait deviation. For the last case, being more complex, the best option is to use inertial sensors with tri-axial accelerometers and gyroscopes (inertial measurement units, IMUs); from a holistic point of view, different sensors will be necessary to provide additional information about the type of DPA. A first approximation to determine activities of daily life (ADLs) is through location (i.e., if the person is in the bathroom early in the morning, it may be associated with toileting, while if he/she is in the kitchen, it would be associated with cooking). Hence, symbolic location (room-level location) does not provide enough information to complement the IMU data. But in other cases, if we only know that one person is in the living room, we will not be able to discern whether he/she is watching TV, eating or wandering around the room. That is why in certain environments a more precise location is needed in order to identify the real activity carried out. In this way, we could say that positioning can indirectly provide valuable information for monitoring DPA and, therefore, contribute to facilitating aging in place.

Nowadays, older people spend most of their time indoors, and indoor location is a current topic of research. The most common approach to localize them with wearable devices is the use of radio frequency (RF) technologies based on received signal strength (RSS). Nevertheless, there are other technologies that may be involved, such as cameras, acoustic signals, radio frequency identification (RFID), ultra-wideband (UWB), visible light, infrared (IR) or other options with different levels of accuracy [13,14,15]. But unfortunately, no standardized indoor localization solution, such as GNSS (Global Navigation Satellite System) for outdoors location, is currently available.

Despite all the technological progresses made in the last decade, the assessment of kinetic parameters at a clinical level is still carried out by restricting the movement to a specific area and to a specific moment in time, e.g., when medical staff checks the patient following one of the standardized clinical scales. Moreover, conventional screening methods for assessing the ability to perform activities of daily living or even the activities performed, are often based on self-reports. We address this limitation by proposing the parameters’ evaluation, namely walking speed and level of PA, during an uninterrupted period of time and in the person’s usual environment.

The goal of this work is to assess the feasibility of objectively measuring the DPA levels of older adults over time. Our work has been developed under two premises: collect data of the older adults while in their usual environment and over a long period of time. Moreover, older adults often lead routine lives, so continuous monitoring of their DPAs will help early detection of the onset of anomalies in these routines that might alert both their caregivers and clinicians to potential physical or cognitive decline.

Our proposal is based on the use of different technologies, namely: IMU, barometer, WiFi for symbolic location and an ultrasonic local positioning system (ULPS) for precise positioning. The multisensory fusion over time will provide objective information on DPA parameters to subsequently generate behavioural routines. However, as the level of precision may differ, different approaches are proposed. That is why this work reports real-world examples with different approaches to evaluate their performance in each situation.

According to our knowledge, there is not a wearable used in digital health applications which could simultaneously provide information about physical activity and accurate positioning of the person, so as to correlate both types of information with the person daily activities. Thus, this work also introduces a wearable designed by the authors, the FrailWear [16], which provides information from an IMU, a barometer and IR and ultrasound sensors (US) for the ULPS, with the aim of monitoring DPA no matter if the individual lives alone or in a nursing home. Therefore, this work addresses different challenges to achieve the main objective of DPA assessment for clinical purpose from three different approaches:

- An initial one monitoring mainly the activity data obtained from and initial system, counting number of steps during active periods of time and step frequency when walking, demonstrating the feasibility of the process for the subsequent generation of behavioural patterns;
- A second approach, where room-level location information is included to infer the type of activity. In addition to the inertial measurements, we would also include symbolic location based on WiFi and a barometer;
- Finally, we would consider precise positioning in selected areas. In this phase, an ad hoc wearable designed for DPA estimation would be introduced, adding precise adding precise positioning to the available inertial information.

The rest of the paper is organized as follows: Section 2 shows a review of the related works; Section 3 introduces an overview of the experimental approaches; Section 4 describes an inertial based approach for activity monitoring; Section 5 and Section 6 cover symbolic location and precise positioning, respectively; Section 7 includes a discussion of the most relevant results; and finally, conclusions and future work are presented in Section 8.

## 2. Related Work

Kim et al. classify recent smart home technologies that monitor older adults’ health, noticing that there is no connection between the home monitoring technologies now available and how they can be integrated into the older people’s homes [3]. An essential feature of home monitoring is the ability to locate the end user. In most scenarios in digital health, location allows inferring the activity of a subject [17]. A multitude of alternatives based on multisensory systems have been proposed for indoor location by combining different sensing technologies and devices, including radio frequency, infrared, tactile and light sensors, which are mounted on furniture and appliances in the environment, or even a prototype of a smart floor [3,18]. However, it is not yet possible to provide accurate and cost-effective long-distance indoor tracking that does not require direct vision and is not dependent on lighting or affected by possible occlusions [19]. In addition, monitoring of older adults will require small, comfortable and low-maintenance devices or wearables. This is the reason why in the literature there are solutions with different technologies for indoor location, such as WiFi, Bluetooth Low Energy (BLE), US or UWB as global systems; or pedestrian dead reckoning (PDR) based on locally referenced inertial systems.. WiFi- and BLE-based systems provide meter-accuracy positioning, or even room-level, while other alternatives such as US and UWB can reach centimetre-accuracy positioning [17]. One of the main limitations of these accurate systems is that they require direct vision, while those based on inertial systems accumulate errors over time, leading to drift in positioning. This explains that the most complete solutions are based on systems which fuse several technologies. According to the compilation work of Shum et al. on indoor location data for tracking human behaviours [17], the most widespread technologies for activity level monitoring are IMU and RFID, while for precise positioning, the most popular is UWB. However, there are no works exploring the possibility of using US, which is an accurate and low-cost alternative.

The large development of technology associated with the Internet of Things (IoT), as well as indoor location-based services (ILBS) and possible alternatives to assist in daily tasks are trending topics, as shown in the review of wearables for the remote monitoring of older adults, especially through physiological parameters, as explored by Olmedo-Aguirre et al. [4] or the study focused on IoT by [5], and the ILBS review as shown in [17]. Considerable research in the literature links PA and different kinematic parameters (such as gait speed) to health benefits in older adults [20,21]. Furthermore, a decline of kinematic parameters has been identified with different negative clinical states such as Parkinson’s disease, dementia [22] or even global degradation through frailty [20,23,24]. Therefore, it is important to control and monitor PA and kinematic parameters to be able to track diseases or even to detect them in early stages. Many studies focus on isolated monitoring of daily activities, but for detecting health and behaviour trends that might signal person’s decline, changes in habits and/or risk or resilience over time, long-term monitoring is more valuable even if is not continuously carried out. Many previous works have studied the physical activity of people for different time frames. Most analyse daily activities for 24 h [23,25] or for a week [26], but only a minority for longer [27,28]. Nowadays, there is a widespread concern for monitoring daily activity with the idea to generate routines [3,6,19]. In addition, this long-term monitoring should be able to be carried out in the usual environment of the people, regardless of whether it is in the city or in rural areas, or if they reside in a nursing home [27] or in their own home [29]. For this last case, the monitoring presents another important advantage because as they lead an independent life, both the monitored people and the families have peace of mind.

Reviewing recent works on the use of wearable devices for measuring PA and kinematic parameters in older adults, we find there is a vast variety of gadgets and parameters, likely due to the lack of standards for the use of these parameters in clinical protocols and the quick technological development [3,8,30]. The most common wearables are accelerometers, health bands or actimeters [26,31]. However, it has been shown that actigraphy overestimates the PA levels, whereas sedentariness is underestimated in older adults [32]. Accelerometer-based DPA monitoring is often performed via IMUs [26,33,34] or smartphones [35,36]. Some works show monitoring results of institutionalized older adults [37], other of people living in their own home [32] and some of them simulate activity of daily living in a lab [33]. Some authors are focusing on approaches for aging-in-place, as it is proposed in reference [27], based on a sensing platform for health monitoring. However, despite the large scientific production developed in this area in recent years, there are still no reliable and objective solutions to quantify DPA as a clinical parameter.

## 3. Experimental Approaches

### 3.1. Physical Activity Monitoring Approaches

One of the aims of this work is to monitor long-term physical activity in older adults using different location-based approaches. The selection of the location system will depend on the type of information that it is required to provide and the environment in which the measurements are carried out, as it has been introduced in the previous section. The goal is to provide objective information that can help clinicians to carry out a more accurate assessment of the person’s state of frailty.

**Figure 1:**
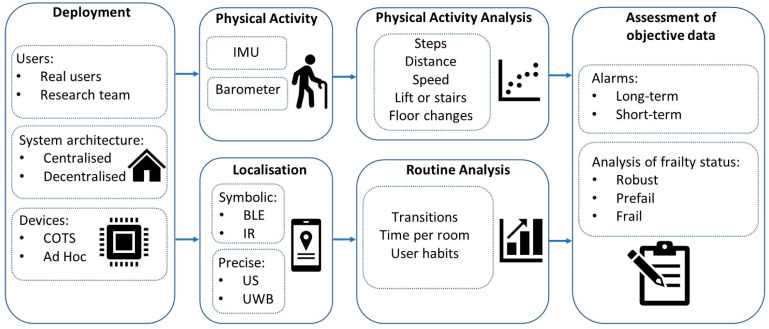
Global overview of the monitoring system.

Figure 1 depicts our view of a complete monitoring system. Our vision includes an adaptable system to different environments (private houses, nursing home, etc.) and not only for research team volunteers that know how to use the technology but for real users. Sometimes we can find a commercial wearable for monitoring PA, but if we demand high performance, it is likely that we will not reach a commercial solution, and we will have to design our ad hoc wearable. In the conceptualization of the system, physical activity monitoring cannot be performed for a short time; long-term analyses are required to provide objective information on the level of frailty. One important aspect is to obtain user’s routines linked to their positioning that can be related to activities of daily living. For this, the system requires the inclusion of sensors for symbolic localization (e.g., BLE, IR) or precise positioning (e.g., UWB, US).

In this paper, we show how to identify DPA by using different sensory systems. Each one can be implemented individually, or they can be fused improving the quality of the monitoring results. We describe below three approaches.

#### 3.1.1. Inertial Sensor-Based Monitoring Approach

The aim is daily activity monitoring over long periods of time using a minimally intrusive wearable such as an IMU, mainly using the integrated accelerometers and gyroscopes. Through the raw data obtained from the sensors, parameters such as number of steps, distance walked and amount of activity are estimated through processing. In addition, if a barometer is added, the measured atmospheric pressure can be related to changes of floor or use of lifts, obtaining other parameters of interest. For this goal, we propose to use a COTS (commercial off-the-shelf) IMU, based on the commercial NGIMU [38], which integrates a barometer in addition to the ordinary sensors.

#### 3.1.2. Symbolic Location-Based Monitoring Approach

In addition to using the physical activity measurement information from the first approach, we propose adding symbolic location. For this, the same multisensory device, NGIMU, is used, as it includes WiFi connectivity. The WiFi connection of the device with routers deployed in rooms of interest and allows for locating a person in a certain room at a specific instant of time. In this way, the time spent in each beaconed room is obtained. This information is useful to detect the monitored person’s routines linked to localization.

#### 3.1.3. Precise Positioning-Based Approach: The FrailWear System

In addition to detecting in which room the person is in, it can be very interesting to know in which room coordinates the person is, as to be able to discriminate whether he/she is at the table, in front of the television, lying in bed, etc. Since there is no COTS device that can be used to obtain all these data, we have designed a wearable device (it is named FrailWear [16]) that includes a multisensory system (IMU, barometer, IR and US technologies). It has been tailor-made to meet all needs for measuring physical activity and precise positioning. It integrates the previous described functionalities, but symbolic positioning is performed by the IR sensor (i.e., instead of a WiFi router, an IR beacon has to be installed in the room of interest which emits a unique code associated with the room). The additional feature of precise positioning is carried out by using US sensors. US beacons have to be deployed in the rooms where it is necessary to know the coordinates where the person is (e.g., it may be of interest to analyse whether the older adult living in a nursing home always sits in the same place to watch television, to get lunch, etc.). With the analysis of all the information obtained by the multisensory system, it is possible to extract the ADLs that each monitored person performs at a semantic level. That is, for example, from 15:00 pm to 16:00 pm he/she is sitting on the sofa watching TV in the living room; or, from 16:00 pm to 17:00 pm he/she is cycling in the gym. This information is useful to analyse routines to monitor how they progress over long periods of time and to detect anomalies that may be indicative of physical or cognitive impairment in older adults.

For testing the different proposals, we have performed some trials in different environments (private homes, nursing home and research laboratories). Some volunteers have participated in them to provide realistic results.

### 3.2. Participants

Four participants were recruited for long-term DPA assessment.

- Two healthy men living in their own home, both 75 years of age, clinically defined as robust/prefrail according to the Fried Frailty Index at the start of the study;
- Two frail women, an 88- and 92-year-old, institutionalized in the nursing home of Albertia, Las Palmeras, in Azuqueca de Henares (Spain). Both women used a walking aid.

Additionally, some researchers, aged between 23 and 29, participated in some of the calibration and validation tests of our proposals.

Guadalajara University Hospital Ethics Committee approved the study protocol (Institutional Review Board No.2018.22.PR, protocol version V.1. dated 21 December 2020), and all participants signed a written informed consent.

### 3.3. Test Environments

The experiments were carried out in different places:

- Preliminary tests were performed in the School of Engineering of the University of Alcalá (Madrid, Spain);
- Other tests were developed in the participants’ home;
- Finally, other experiments took place in a nursing home with approximately 100 residents. The residence has a total of four floors with approximately 2875 m^2^ per floor. Each floor has different facilities, which are specified in Table 1.

## 4. Inertial Sensor-Based Monitoring Approach

As was introduced, this section describes the approach used to measure DPA with only inertial-based commercial systems, focusing on both the required instrumentation, and developed algorithms in Matlab 2021b ©.

### 4.1. System Description

**Figure 2:**
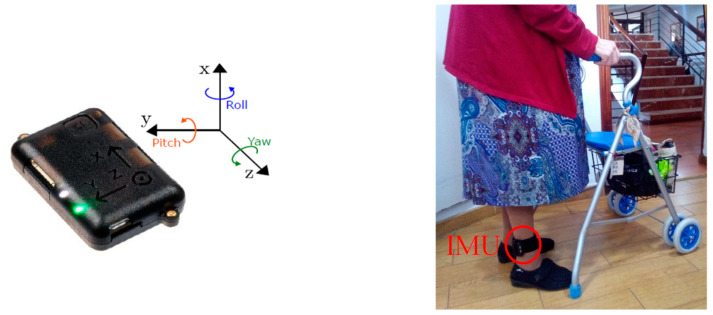
Picture of the NGIMU (**left**) and placement on the volunteer’s ankle (**right**).

**Figure 3:**
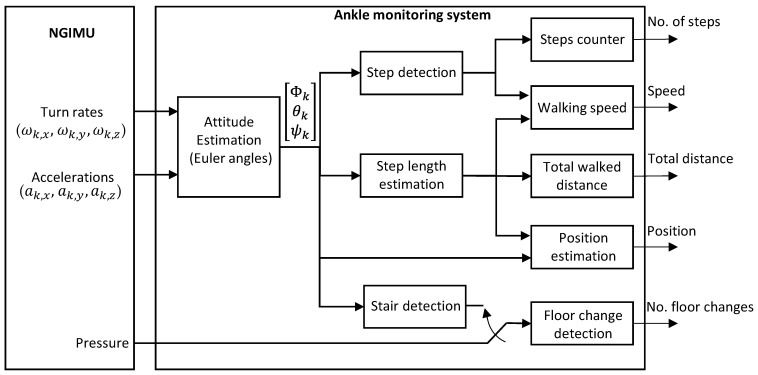
IMU-based ankle monitoring system. This system processes the turn rates, accelerations and atmospheric pressure provided by the IMU, and it is able to generate the number of steps walked by the person, walking speed, walking distance, position and floor changes.

From the point of view of the hardware, the system is based on the commercial wearable named NGIMU [38]. It is a compact data acquisition platform that combines integrated sensors with data processing algorithms and a wide range of communication interfaces, which is very useful for real-time and data-storage applications. We use for this approach the 9-axis IMU, composed of three gyroscopes and three accelerometers (one sensor per axis) and a barometer for atmospheric pressure estimation. The 3-axis magnetometer is not used in this case. The device can be carried out either inside the pocket or placed on the person’s ankle, although for our experiments we have chosen the latter. The appropriate processing of the inertial measurements provides the Euler angles (roll, pitch and yaw) that will be used for the activity monitoring. Figure 2 shows a picture of this sensor and its placement on the volunteer’s ankle, and Figure 3 shows a block diagram of this ankle monitoring system.

### 4.2. Algorithms

The proposed IMU-based monitoring system (see Figure 3) includes several blocks for providing physical activity measurements, i.e., number of steps, walking speed, position and number of floor changes. The *Attitude estimation* block calculates the Euler angles (roll, $\Phi_{k}$; pitch, $\theta_{k}$; and yaw or heading, $\psi_{k}$) after processing the measurements of accelerations in m/s^2^ ($a_{k,x},$ $a_{k,y},\;a_{k,z}$) and turn rates in °/s ($\omega_{k,x},$ $\omega_{k,y},\;\omega_{k,z}$) for the three axes at every time instant *k* (in this case the sampling frequency is 100 Hz, what means an updated time of 10 ms, enough for our application). Euler angles are estimated by using an extended Kalman filter (EKF) introduced in [39]. The EKF minimizes the effect of noise and artifacts when calculating the Euler angles. The correction stage of the filter is applied when the linear acceleration corresponds to the gravity acceleration, which is the time instant when the foot is on the floor. This work focuses mainly on three algorithms: step detection, step length estimation and floor-change detection.

#### 4.2.1. Step Detection

The algorithm estimates the number of steps the volunteers walked by processing the Euler pitch angle $\theta_{k}$. Once the pitch angle is estimated from the EKF, the number of steps can be determined by the zero-crossing technique (ZCT). This technique is used to estimate the local maximum and minimum of the pitch signal and, from a certain amplitude threshold, to determine if it is a step. Algorithm 1 shows the algorithm for detecting a step.
**Algorithm 1:** Step detection algorithm   **Inputs**: pitch    **Outputs**: n_steps    n_steps = 0    max_processed = inf    min_processed = −inf    **if**(k > 100) **then**
      mean_pitch(k) = mean(pitch(k − 100:k))       pitch_processed(k) = pitch(k) − mean_pitch(k)       **if** pitch_processed(k − 1)<0 && pitch_processed(k) > 0 **then**
         n_ZC = n_ZC + 1          up = 1          down = 0       **if** pitch_processed(k − 1) > 0 && pitch_processed(k) < 0 **then**
         n_ZC = n_ZC + 1          up = 0          down = 1       **if** up == 1 && pitch_processed(k) > max_ processed **then**
         max_processed = pitch_processed(k)       **if** down == 1 && pitch_processed(k) < min_ processed **then**
         min_ processed = pitch_processed(k)       **if** n_ZC == 2 **then**         **if abs**(max_processed − min_processed) > 25 **then**              **if** max_processed > 7 && min_processed < −7 **then**                    n_steps = n_steps + 1                    n_ZC = 0 

#### 4.2.2. Step Length Estimation

After a new step is detected, the step length $SL_{k}$ is estimated from the absolute maximum and minimum of the pitch signal, according to (1) and (2). The parameters *a_h_* and *b_h_* connect the pitch angle amplitude to the step length through a linear relationship, and they can be calculated by the universal regression proposed in [40], or experimentally. In this case, they are obtained experimentally, easily using a reduced set of measurements, and resulting in *a_h_* = 0.0294 and *b_h_* = 0.232. The position and orientation ($x_{k},y_{k},\;\psi_{k}$) derived from only the IMU measurements are computed according to (3).

(1) $$\Delta \theta_{k}=\theta_{max}-\theta_{min}$$

(2) $$SL_{K}=a_{h}\cdot \Delta \theta_{k}+b_{h}$$

(3) $$\left[\begin{array}{c} x_{k}\\ y_{k}\\ \;\psi_{k} \end{array}\right]=\left[\begin{array}{c} x_{k-1}+SL_{K}\cdot \cos\psi_{k}\\ y_{k-1}+SL_{K}\cdot \sin\psi_{k}\\ \psi_{k-1}+\Delta \psi \end{array}\right]$$

#### 4.2.3. Floor-Change Detection

The objective of the algorithm is to detect when the person changes between floors inside the building. Although the pitch angle processing can detect the used of stairs, as the person can use both the stairs and the lift to move between floors, we have used the barometer information to detect the floor changes. To identify a pressure variation as a floor change, it is required a previous calibration to define thresholds. Thus, the detected pressure changes are compared with semiempirical thresholds to know the number of floors that have been climbed up or down, depending on whether the pressure increase is negative or positive, respectively.

Through the described data processing algorithms, we detect walking patterns and classify the activity into three categories: standing/sitting, lying and walking. To describe the DPA patterns, the following parameters were calculated: daily cumulative walking duration (in hours), average walking speed, and for the institutionalized volunteers, the number of floor changes per day.

### 4.3. Experiments for Monitoring Physical Activity over Time

The volunteers carried out the previously described IMU for several days (between 5 and 25 days), around 8–10 h a day (from they wake up until the battery runs out of power) and we repeated the tests five times in 1.5 years. Depending on the volunteer, then number of days and the gap between test days was different, as it is described below.

#### 4.3.1. Short-Term Monitoring

**Figure 4:**
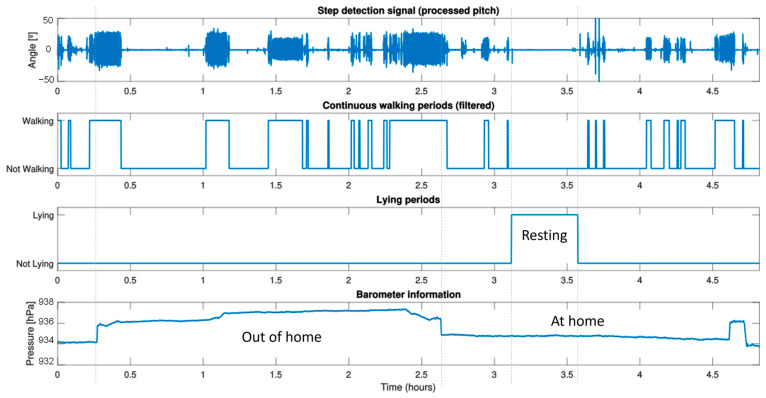
Intraday results of a prefrail volunteer. In the figure, from top to bottom: (1) step detection through the pitch signal; (2) the result of processing the pitch signal to detect continuous walking periods; (3) rest time; (4) the value of the atmospheric pressure over time that helps to know whether the person is at home or not.

Figure 4 shows the results for only 5 h monitoring of a prefrail volunteer living in his private home. These results provide the care staff with information about the periods of the day when the volunteer is continuously walking, the walking speed of that period, its duration and pressure changes. In this case, this volunteer lives in a four-story building, and thanks to the barometer, it is possible to know when he leaves and enters the building.

**Figure 5:**
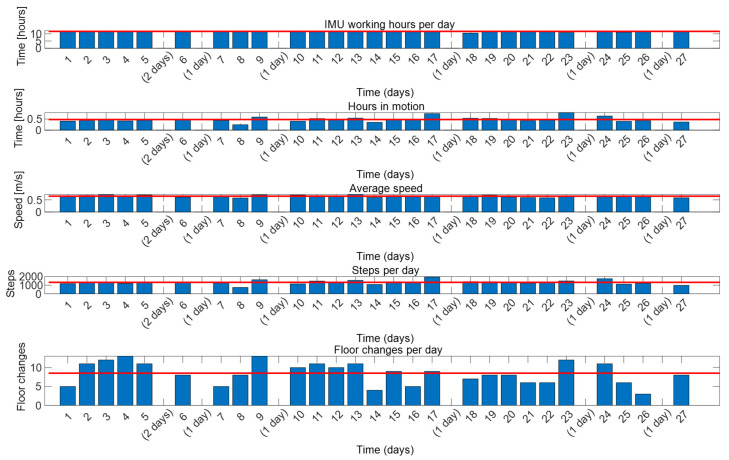
Daily results of a frail volunteer for 27 days. In the figure, from top to bottom: (1) number of hours that the volunteer carries the IMU; (2) number of hours in motion; (3) average walking speed; (4) number of steps per day; (5) number of floor changes per day.

If the person is monitored every day, then in processing the daily data of Figure 4, we can calculate the DPA. Figure 5 depicts a daily summary of a frail volunteer for a 27-day test that shows the volunteer’s trend in PA, which is very useful for generating routines. This volunteer lives in the described nursing home and is a 92-year-old woman. The test includes the number of hours when the IMU was active, the number of hours when the volunteer was in motion, the average walking speed, the number of steps and the number of floor changes. It is indicated in the graphs the days without information (blank days), as the volunteer did not wear the IMU (either she left the nursing home, or the battery of the device was discharged). On average (the red line in each graph indicates the average value of each parameter during the test), the volunteer wore the IMU 11.65 h a day; she was active, i.e., walking, 0.48 h per day; her walking speed was 0.65 m/s performing around 1335 steps a day; and she changed floors 8.5 times per day (the volunteer not only changed floors for meals, but also for visiting the gym). Although the walking distance is not included in the figure, it can be easily calculated by using Equation (2). In the example of Figure 5, the time in motion, the average speed, and the number of steps of the volunteer related to the day 8 is lower than the average values (blue line). The cause of this reduction in her usual activity was an upset stomach. This fact was later confirmed by the medical staff.

**Figure 6:**
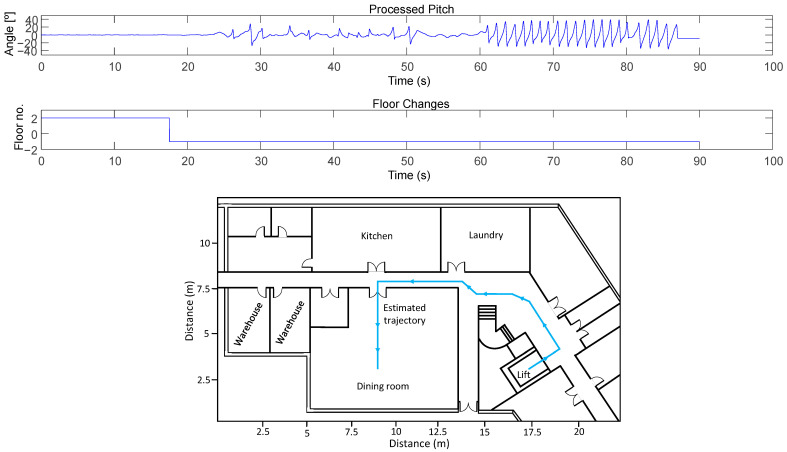
Example of trajectory performed by the volunteer from the lift (second floor) to the dining room (floor −1). The walk took place in the morning, when the volunteer headed to the dining room for breakfast.

The IMU-based ankle monitoring system can estimate the volunteer’s trajectory (see Equation (3)). Figure 6 shows an example of the trajectory (adapted to the known map with the match-mapping technique) when the volunteer walked from the elevator on the second floor to the dining room on floor −1. During this period (around 90 s), the volunteer walked a total distance of 18.8 m and a total of 21 steps. As the figure shows, the volunteer started walking 40 s after exiting the lift (maybe there were more people inside the lift).

#### 4.3.2. Long-Term Monitoring

To observe the long-term evolution of the users, the statistical results obtained from different short-term staggered tests carried out over time (from several months to one and half years) are analysed (five tests in total). This long-term monitoring can be useful to detect trends related to the physical activity of the volunteers that could be related to the physical deterioration of the monitored older adults. Table 2, Table 3 and Table 4 show the statistical results (average, Avg, and standard deviation, SD) related to the evaluated parameters of three volunteers (robust, prefrail and frail). The number of monitored days of each test is also indicated below the test labels (i.e., T1 (20 d)), the minimum being 5 days, which is representative enough considering that their days are very routine. The number of elapsed days between tests is also indicated, and for the data shown in Table 2, it changes from 58 days between the first and second timeframe to 365 days between the second and third.

**Figure 7:**
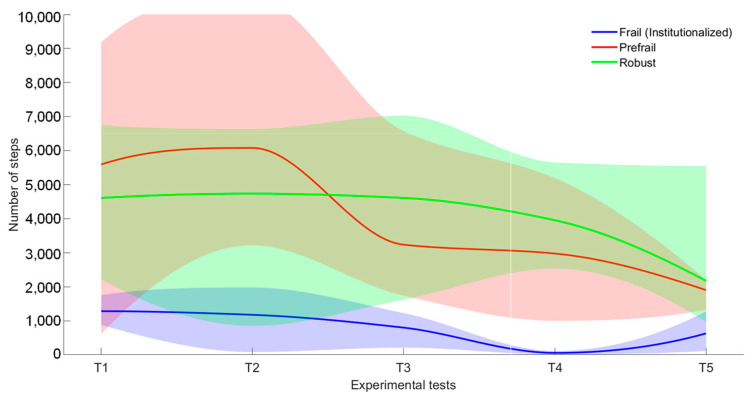
Number of steps (mean, maximum and minimum): evolution results of the volunteers (frail in green; prefrail in red and robust in green).

**Figure 8:**
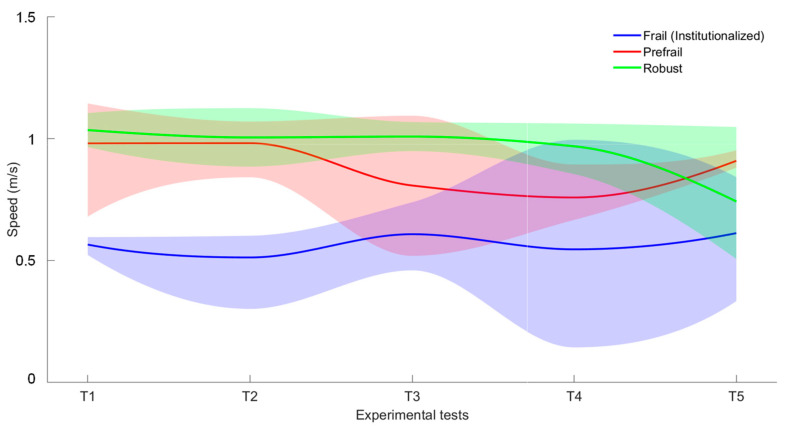
Walking speed (mean, maximum and minimum): evolution results of the volunteers (frail in green; prefrail in red and robust in green).

To better evaluate the volunteers’ physical activity, Figure 7 and Figure 8 show the evolution of the number of steps and walking speed (mean, maximum and minimum) including all tests, respectively.

It can be observed that the mean number of steps (see Figure 7) per each test of the robust user (in green) is constant during T1 to T4, but in the last test the activity is considerably reduced (this was correlated with an ankle problem). In case of the prefrail volunteer (in red), there is a significant decrease of more than 2000 steps per day due to a deterioration in the health condition of this user according to the medical staff. The lowest values are matched to the frail volunteer (in blue), showing an average value lower than 1500 steps per test. Furthermore, it is interesting that T4 shows very low physical activity for the frail volunteer. Medical staff informed that it was due to a previous user’s fall. Nonetheless, as T5 indicates, she recovered most of her activity with more than 600 steps per day on average after the accident. It is worth mentioning that the standard deviation also seems to decrease with the number of steps, being more stable for the frailest volunteers. Indeed, these volunteers are more routine.

For the walking speed (Figure 8), the robust volunteer shows a constant value of 1 m/s for all tests except for the last one (T5), due to the mentioned ankle problem. The prefrail volunteer started in the first test with the same value of walking speed than the robust user. However, as the evolution of the number of steps, this volunteer demonstrated a quite significant decrease of 0.2 m/s in his walking speed, so his state of frailty has worsened over time. Although there seems to be an improvement in the last period evaluated, this is circumstantial as the decline continues according to subsequent medical data, in line with the trend observed from the data evaluated from DPA. Finally, the frail volunteer had a low walking speed (around 0.55 m/s) but one that was constant over the tests, including T4 after the accident.

## 5. Symbolic Location-Based Monitoring Approach

As was described, the information about DPA can be enriched if it is added symbolic localization. As the previously tested multisensory device, NGIMU [38], includes WiFi connectivity, we propose to deploy WiFi access points in the rooms of interest. Then, we will improve the PA monitoring including where the person is performing the activity. We introduce in this section a proof of concept to show the viability of using the NGIMU for symbolic localization.

### 5.1. System Description

**Figure 9:**
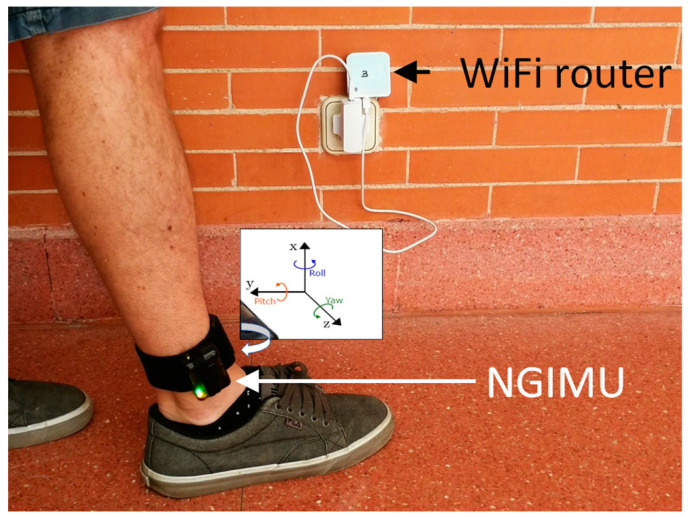
NGIMU located on a person’s ankle and WiFi router.

When using the NGIMU WiFi communication interface, it can work as a client or as an access point (reception system). In this case, we propose to use it as an access point that is able to record a configured WiFi service set identifier (SSID). The information that the NGIMU provides is the percentage of connection with the SSID, between 0 and 100%. For this functionality, it is required a WiFi emitter in the room, such as the short-range commercial WiFi router (TL-WR802N) [41]. It has a transfer rate of 300 Mbps, and its broadcast frequency is between 2.4 and 2.4835 GHz. This device is configured as router, broadcasting a specific SSID, that has to match with the one defined for the NGIMU. Figure 9 shows both devices in operation.

### 5.2. Symbolic Localization

The objective is to obtain the volunteer’s location with room-level granularity. A router is installed in each one of the most frequented rooms, representative of the person’s activity. All the routers are then configured with the same SSID previously defined in the NGIMU. The presence in a particular room is inferred when the wearable connection percentage (%C) is higher than empirical threshold (Th). The higher the percentage, the closer the NGIMU is to the emitter. In this case, we do not need to know the coordinates of the positioning, but just whether the person is or not in the room. We have defined the threshold considering the characteristics of the room (size, obstacles between emitter and receiver, etc.). 

**Figure 10:**
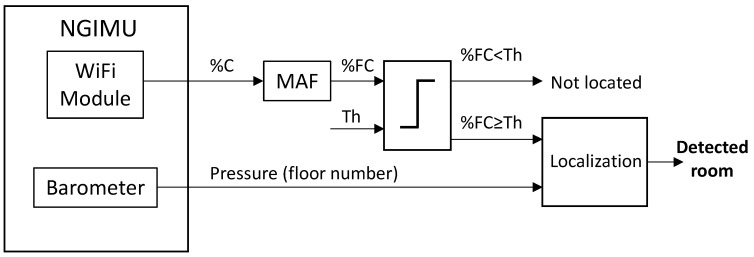
Block diagram of symbolic localization algorithm.

Figure 10 shows the block diagram of the symbolic localization algorithm. The percentage of connection provided by the NGIMU is smoothed with a moving average filter (MAF), obtaining a filtered connection, %FC. When %FC is higher than the threshold, it is considered the pressure information to know in which floor the person is, as all the routers broadcast the same SSID. The result is the room where the user is.

### 5.3. Experimental Results

**Figure 11:**
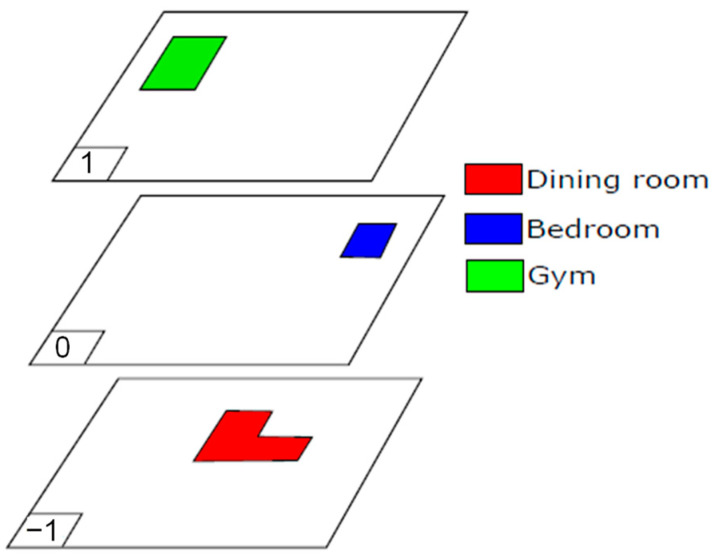
Selected rooms in the different floors of the nursing home where a router WiFi is placed. (Colour code: dining room, in red; bedroom, in blue; gym, in green).

We tested this approach in the nursing home and selected the following relevant rooms for a volunteer: bedroom, dining room and gym. As is indicated in Table 1, these rooms are on different floors. Figure 11 outlines the locations of the rooms on each floor. We have assigned a colour code for distinguishing them in the experiments.

**Figure 12:**
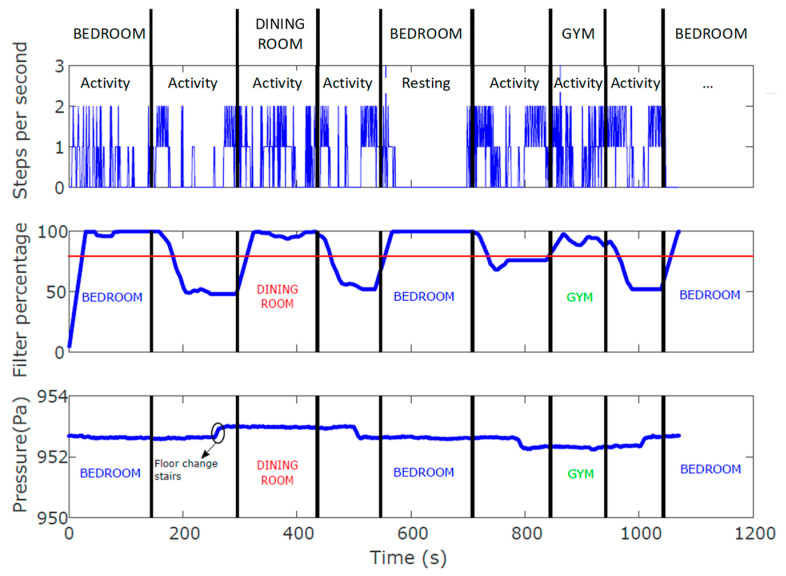
From the top to bottom in figure: steps per second, percentage of the WiFi signal and pressure values (note: labels on the top of the figure indicate the ground truth).

We carried out a first test to know the percentage of WiFi connection in each room. We followed a pre-established routine with a member of the research team in order to validate the proposal knowing the ground truth. Figure 12 shows the number of steps per second of the volunteer, the percentage of the WiFi signal and the pressure values during a route of more than 1000 s. The black lines represent a change in room according to the ground truth (labels on the top of figure) and the different rooms are identified according to the colour criteria shown in Figure 11. It can be observed that the received percentage of the WiFi signal is quite close to the maximum (100%) when the user is located on the monitored rooms, so it is possible to know approximately the time spent by the user in each room. After repeating several times this test, the threshold was fixed at 80% (Th = 80%, red line in Figure 12) to conclude when the user was in the room. The symbolic localization success rate was 95.6%, enough to our application.

**Figure 13:**
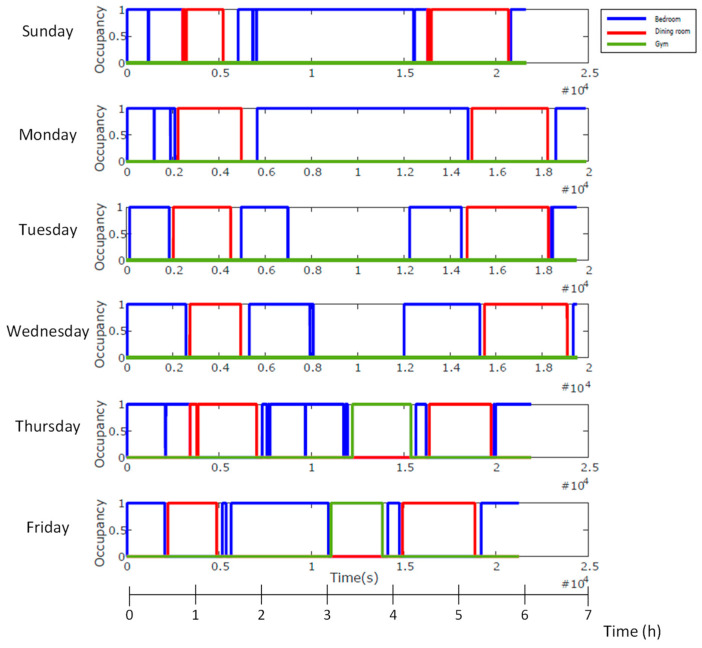
Occupation intervals of a frail volunteer in each room for 6 mornings (from Sunday to Friday—each row represents every day).

Once the algorithms were validated, other tests were carried out with institutionalized people during several days. As an example, Figure 13 shows the results of 6 mornings (from Sunday to Friday, around 6–7 h per day), representing the occupation intervals of the selected rooms. By associating an activity to each room, one could infer the volunteer’s breakfast and lunch periods or rehabilitation in the gym (Thursday and Friday), etc. These results were consistent with the usual volunteer’s schedules provided by the caregivers.

## 6. Precise Positioning-Based Approach: The FrailWear System

The previous results have shown the potential of DPA tracking and symbolic localization for localization-based routine generation. However, we can go a step further, not only providing symbolic localization, but also precise positioning. Although precise positioning is not a demand for physical activity monitoring, it can be very interesting when it is considered the additional objective to detect user’s routine. If we monitor institutionalized people, it is very common that they sit in the same place in the dining room for meals or in the living room for watching television. If we are able to detect that the monitored person changes his/her positioning over time when doing those social activities, we can produce a valuable information for the carers that might be the onset of a cognitive impairment, e.g., Alzheimer’s disease. Based on this premise, we have developed our own ankle monitoring system which incorporates the functionality of indoor precise positioning, by using ultrasonic local positioning system (ULPS). We call it FrailWear. The FrailWear hardware was first described in [17]. This section briefly introduces this system and shows some experiments that prove its feasibility for precise positioning-based routine generation.

### 6.1. The Global FrailWear System

**Figure 14:**
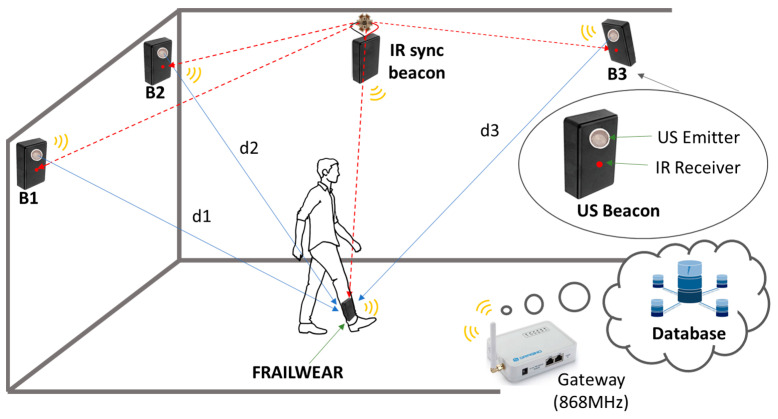
Global FrailWear system, including external ULPS and infrared link [16].

The global system consists of an external infrastructure and the FrailWear device. The external infrastructure is based on an ULPS that provides precise positioning (centimetric accuracy) and an infrared module, used to synchronize the whole ultrasonic positioning system and to provide symbolic localization at a room level (as the previously describe WiFi system in Section 5). All the information is stored either on a SD card or on the cloud through a LoRaWAN Network architecture 868 MHz gateway. Figure 14 shows an overview of the global system.

**Figure 15:**
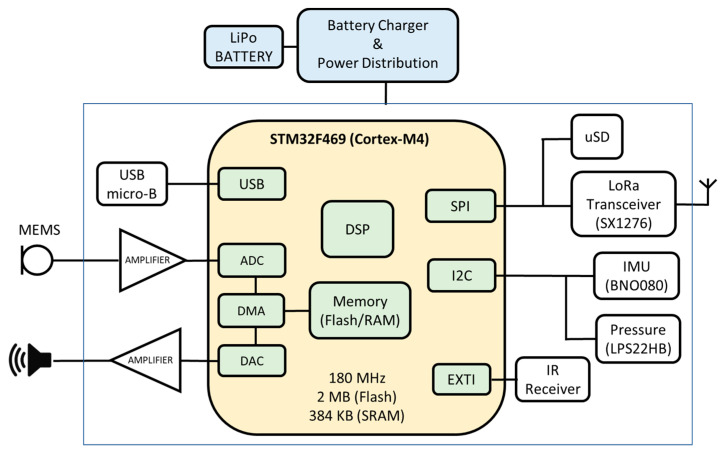
Block diagram of the FrailWear device [16].

The FrailWear device is a wearable multi-sensory system (see Figure 15) and consists of different blocks. The most relevant subsystems are briefly described below (more information is available at [16]):

- The main core of the device is a Cortex-M4 processor (STMicroelectronics, 32-bit STM32F469 [42]), which reads through I2C (Inter-Integrated Circuit), and SPI (Serial Peripheral Interface) buses the information of digital sensors. It is particularly distinct in its capability to acquire the ultrasonic signals from the encoded beacons (with Kasami codes) through a microphone, to decode the information and to process it to autonomously to provide 3D positioning. Algorithm 2 shows the US-based localization algorithm (more details about how our ULPS works can be found in [39]);
- It incorporates an IMU subsystem, implemented on a dedicated board using a Bosch’s BNO080 sensor [43]. This sensor integrates a 3-axis accelerometer, gyroscope and magnetometer, packaged with an ARM Cortex M0+ microcontroller that allows to implement high-level algorithms to reduce the computation load of the main microcontroller;
- The barometer utilized is a high-resolution MEMS nano pressure sensor with absolute digital output (STMicroelectronics, LPS22HB [44]) and an accuracy of ±0.1 hPa, which is equivalent to about 10 cm in vertical resolution. Additionally, the chip has an integrated temperature sensor with a 12-bit resolution that provides an accuracy of 1.5 °C. This temperature value is then used to obtain higher accuracy when calculating the ToF (Time-of-Flight) of the ultrasonic signals for the precise positioning.

**Algorithm 2:** US-based localization algorithm   **Inputs**: int_IR //infrared signal synchronization    **Outputs**: x, y, z //3D positioning    kasami_code = load_kasami_codes()    f = 100e3 // Sample frequency    v = calculate_sound_speed(temperature) // Sound speed    sequence_samples = 1224    **if** (int_IR == 1) **then**       room_ID = decode_IR_code()       US_signal = acquire_US_buffer()       pos_US_beacons = load_US_beacons(room_ID)       **for** i=1:n_US_beacons          corr(i) = correlation(US_signal, kasami_code(i))          pos_peak(i) = peak_detection(corr(i))          distances(i) = (pos_peak(i)- sequence_samples)*v/f       x, y, z = Gauss_Newton(distances, pos_US_beacons)

- The infrared module is based on the TSOP7000 infrared receiver from Vishay [45] and works at a 455 kHz carrier frequency. It is used to synchronize the whole ultrasonic positioning system. To obtain a symbolic location at a room-level accuracy, the signal emitted by the synchronization beacon is encoded with an 8-bit code. This allows the room where the person is located to be uniquely differentiated as the infrared signal is confined within the walls. By default, the IR beacons are configured with an emission frequency of 1 Hz, i.e., they emit both the synchronism pulse and the room identification code every second (it is required that the person stays in the room more than one second to be identified);
- The ultrasonic signals emitted by the beacons are acquired with a MEMS microphone SPU0414HR5H-SB [46] followed by a built-in amplification and high-pass filtering stage previous to an analog-to-digital converter (ADC). The microphone is mounted on an external board to place it on a body location where the best ultrasonic coverage is achieved;
- The system is continuously recording a log of all the parameters obtained on a microSD memory card for further analysis, although it also has a LoRa communication port so that data can be transmitted to the cloud even in low coverage environments with long-range, low-power wireless communication (this feature is not used in the current work).

### 6.2. Experimental Results

Several tests were carried out at the School of Engineering of the University of Alcalá to verify the correct operation of the FrailWear system. The tests were performed in three rooms located on three different floors, thus simulating an environment similar to the one described in the nursing home (see Section 4 and Section 5) where the long-term tests were carried out.

In each of the rooms there is an IR beacon which emits a unique code that unequivocally identifies the room, allowing a symbolic location, with the same objective as the WiFi approach describe in Section 5. The rooms are named with a number according to the floor they are on (so room #1 is on the first floor and so on for the three rooms). In addition, 6 ultrasonic beacons were deployed in room #2, as part of the ULPS used for the precise positioning of the volunteer, as depicted in Figure 14. This system can be deployed in common areas where an analysis of the user’s precise position is required (dining room, TV room, etc.). For the validation test, a volunteer wore the FrailWear system on the right ankle, while moving between rooms for approximately 20 min. During the test, the volunteer walked normally through the different rooms, using the stairs and elevator to change floors. The volunteer visited the rooms following this order: room #3→room #2→room #1→room #3.

**Figure 16:**
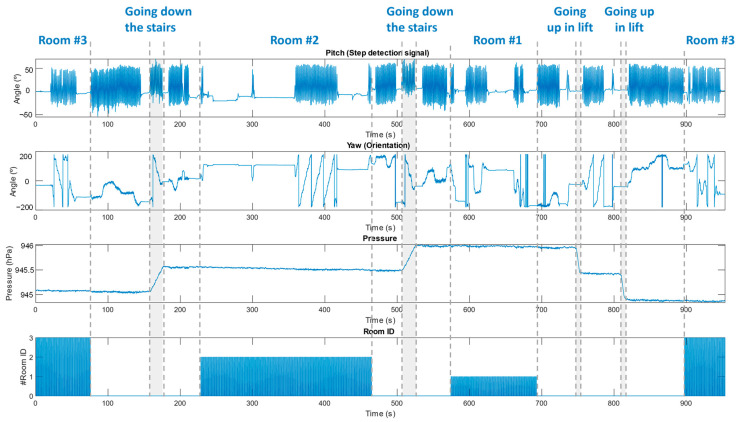
FrailWear results of walking through the controlled environment between rooms #1, #2 and #3 on different floors. In the figure, from top to bottom: (1) pitch signal for step detection; (2) yaw angle (orientation); (3) atmospheric pressure, for detecting floor changes; (4) room identification number (1, 2 or 3).

Figure 16 shows the results obtained from the pitch angle for step identification, the yaw angle for orientation, the atmospheric pressure variation for detecting floor changes and the room index emitted by the IR beacon (ID 1 for room #1, 2 for #2 and 3 for #3). The pitch angle processing provides the same information described in Section 5 for the NGIMU.

**Figure 17:**
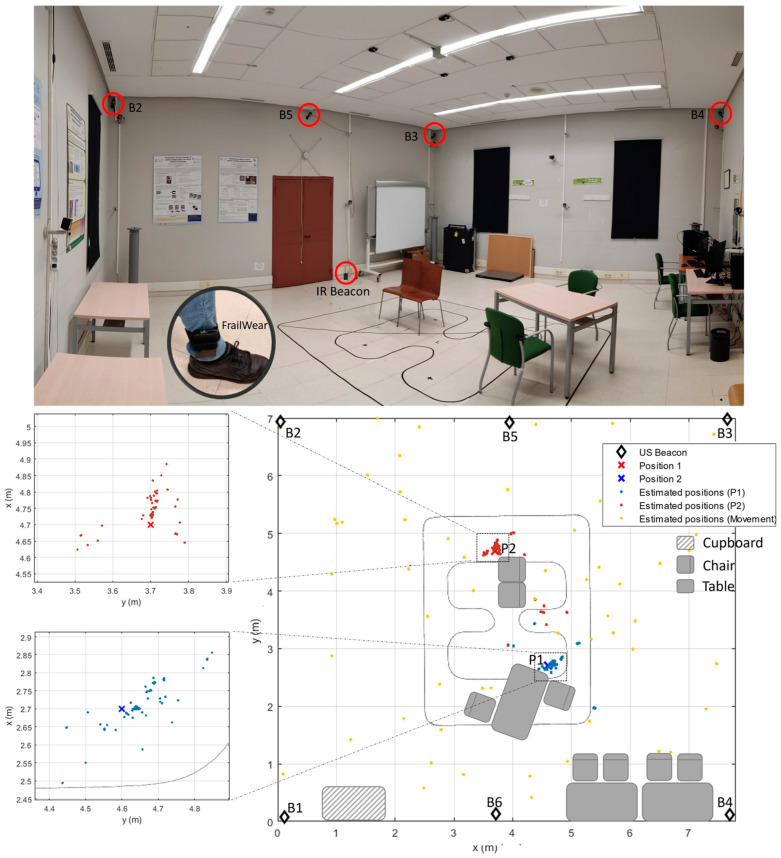
FrailWear positioning results in room #2 with the ULPS. (Top: photograph of room #2 and US sensors location; bottom right: sketch of room #2 with the positioning results superimposed; bottom left: detail of the positioning results in positions P1 and P2).

For showing the capabilities of the FrailWear proposal for precise positioning, we have simulated in room #2 a living room (with some chairs and tables, as the top picture of Figure 17 shows). When the volunteer visited room #2, in addition to walking around the room for 2 min, two 10 min sitting trials were conducted in positions P1 and P2, respectively (see graph at the bottom of Figure 17). In the first position, P1, the chair is in front of a table simulating the position when the person eats one of their daily meals. In the second position, P2, the chairs face the wall emulating that the person is watching TV. In this test, when the volunteer enters room #2, FrailWear receives the IR signal with the room identification code that also serves as synchronism for the ULPS to provide the precise positioning.

**Figure 18:**
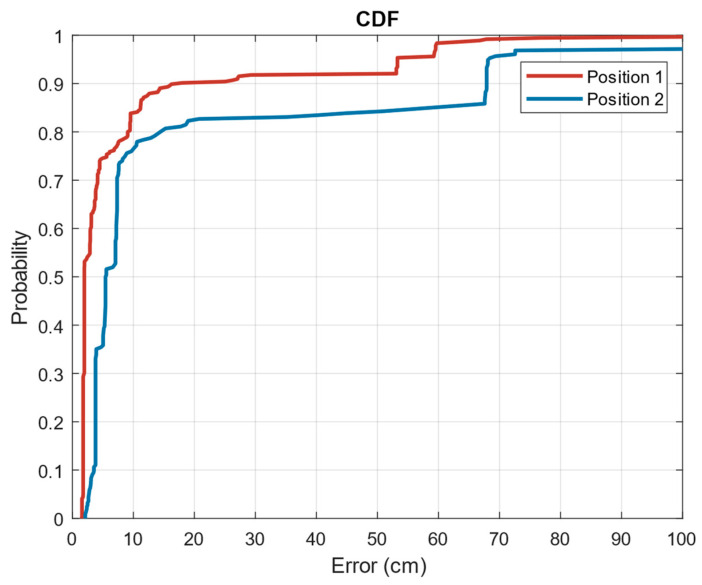
CDF of the positioning results in room #2 with the ULPS.

The results of the ULPS positioning are shown in Figure 17 (graph at the bottom right), where the points obtained with the physical positioning algorithm are shown superimposed on the sketch of the environment. Three different actions can be inferred from the graph: it is highlighted in blue when the person is sitting in position P1, in red in position P2 and in yellow when the volunteer is wandering around the room. Analysing the point clouds obtained in positions P1 and P2 (highlighted in the figure), it can be concluded that the positioning is accurate although there is a loss of information, especially for P1, probably due to the lack of direct vision between the beacons and the sensor shielded by adjacent objects or by the volunteer himself. It should not be forgotten that the sensor is located on the ankle, which provides great accuracy in the estimation of steps with the IMU but limited vision for positioning with the ULPS. To quantify the positioning errors, we have obtained the cumulative distribution function (CDF) for both positions, P1 and P2, when the volunteer spent 10 min at each one. Figure 18 shows both CDF, concluding that for P1 we obtain an error lower than 20 cm for 90% of the cases, and 60 cm for P2.

## 7. Discussion

Human activity monitoring is a complex issue as can be inferred from the present work. Tracking exclusively from inertial systems provides very valuable information that would allow both short- and long-term monitoring of standard parameters such as the number of steps or walking speed, as demonstrated by the data in Figure 7 and Figure 8, which provide some quantification of physical activity. Some studies demonstrated how variations in these parameters can be associated with physiological changes in volunteers and, therefore, could serve as a reference for the generation of patterns. The associated standard deviation of parameters is as important as the mean value since it provides information on variability. This means that a significant increase can also be an indicator that routines are changing and thus can be used to generate an alarm for prompt clinical evaluation. The advantage of using IMUs is that they are low-cost and small systems and, therefore, it is easy to integrate them into the older adult garments in a comfortable and minimally intrusive way.

However, these same results also reveal that such information is not enough for generating behavioural patterns and that it is necessary to include information about the person’s localization. In the case of older adults, who tend to maintain a very routine life and spend a large part of the day at home, indoor monitoring is essential. In many cases a symbolic localization is enough, since an activity can be associated with a location, such as the bathroom. The same applies when there are other sources of information, such as the IMU, that allows inferring whether the volunteer is resting or moving, in order to be able to associate a specific ADL while in a room. This is the case illustrated in Figure 12, where the symbolic location places the volunteer in different rooms, such as in the bedroom on several occasions. But when integrating the IMU results, it is possible to infer whether the volunteer is resting in the room or doing some activity (e.g., tidying the room, etc.). This also helps to analyse whether the person’s lifestyle is too sedentary or not.

There are other situations where the room may be associated with multiple actions or where there may be interaction with other people. In this cases, precise positioning is required to define an ADL. In this sense, the developed FrailWear wearable could be an alternative, as derived from the results in Section 6. It has been demonstrated how the FrailWear IMU provides results comparable to other commercial systems and at the same time, integrates the possibility of both symbolic localization and precise positioning, as required. Figure 16, Figure 17 and Figure 18 compile the results that validate that FrailWear obtains sufficiently detailed information for reliable and accurate tracking to subsequently generate behavioural patterns.

The proposed precise positioning, being multiuser, will not only allow estimation of physical activity patterns, but can also yield information on social activity with other monitored volunteers. The proposed ULPS provides good accuracy, obtaining in the worst-case errors lower than 60 cm for 90% of the measurements, as shown in the CDF in Figure 13. However, it suffers from severe limitations due to signal occlusion between transmitter and receiver. This is evident in the evaluation of positions 1 and 2, although it is possible to distinguish without doubt where the volunteer is. Therefore, if this position is associated to the dining room chair, it could be associated to the action of eating; or if it is associated to a sofa in front of the TV, to watching TV. Less error is obtained for the location when the volunteer is moving around the room. We could infer information about his/her ability to move from an in-depth analysis of the trajectories.

Although the proposed US system for precise positioning provides acceptable results, other more appropriate technologies need to be studied which better adjust to the variability of the environment and which are independent of the position of the sensor.

According to the results, the most suitable system for monitoring DPA in older adults is the one that includes precise positioning (the FrailWear system). The main advantage of FrailWear is its ability to determine DPA with high reliability and completeness, providing quantification of specific parameters such as gait speed, walking distance, etc., as well as its positioning. However, it is a prototype, and its size makes it uncomfortable to carry for a prolonged time on a daily routine. Another drawback of this system is the need to deploy beacons, which is often a problem in private homes.

Although the results are very promising for the person’s routine detection, it is difficult to compare them with what other proposals may achieve, as the technologies, the working environments, the target population and the objectives reported in the literature partially coincide with those indicated in this paper.

## 8. Conclusions

This work has shown three different approaches to identify DPA that can be used to subsequently generate a behaviour pattern. The first one is exclusively based on inertial measurements with an IMU, the second one includes symbolic localization, and the last one also integrates precise positioning for specific areas. Real experiments have confirmed that the third one provides a more complete DPA monitoring. For that, an ad hoc multisensory wearable, called FrailWear, has been developed. This device allows for estimation of PA through an IMU and performs both IR-based symbolic localization and US-based precise positioning. Additionally, it integrates a thermometer, a barometer and LoRa connectivity. It has also demonstrated that continuous older adults’ activity monitoring, with an appropriate IMU placed on the ankle, can provide objective information related to the daily activity of the user, being able to detect anomalies in the physical activity. It can be observed how it is possible to track precisely and objectively the activity of the volunteers, being able to distinguish the change in floors, whether they are using the stairs or the lift, or their presence in different rooms, and if they have been standing or walking around, and their precise positioning in the room can even be obtained (when using FrailWear).

These results show how the monitoring of DPA with a sensor like FrailWear could be the solution to obtain short- and long-term accurate and objective information, allowing the medical staff to react in time with appropriate decisions and to assess the success of DPA-based treatments. This would not only improve people quality of life, but also it would help them to age in place.

Although experimental results have shown the feasibility of the proposal, further experiments are needed, including testing other technologies that can provide more accurate positioning, such as UWB.

## Figures and Tables

**Table 1:** Distribution of the facilities in the nursing home.

Floor	Facilities
−1	2 living rooms, chapel, dining room and garden
0	20 bedrooms, living room with TV and terrace
1	27 bedrooms, terrace, living room and gym
2	18 bedrooms and living room

**Table 2:** Statistical results of the robust volunteer for each short-term test. Tx indicates the number of test (x = 1:5). The number of monitoring days is shown in brackets below the test label (i.e., T2 (14 d)). The days between the tests has also been included (i.e., the elapsed time between T1 and T2 was 58 days, 58 d).

			58 d				365 d				120 d				15 d			
	T1 (20 d)			T2 (14 d)				T3 (11 d)				T4 (5 d)				T5 (5 d)		
Parameter	Avg	SD		Avg		SD		Avg		SD		Avg		SD		Avg		SD
Walking hours	0.81	0.30		0.92		0.45		1.34		0.39		1.00		0.34		0.58		0.47
Sitting hours	9.00	1.45		8.95		1.41		10.62		1.45		11.30		1.48		11.52		0.33
Lying hours	3.00	1.08		2.69		1.61		2.50		1.06		1.13		1.24		0.95		0.45
Steps per day	4611	1354		4737		1863		4612		1619		3954		1320		2174		1932
Speed (m/s)	1.03	0.07		1.00		0.12		1.00		0.06		0.97		0.08		0.75		0.22

**Table 3:** Statistical results of the prefrail volunteer for each short-term test. Tx indicates the number of test (x = 1:5). The number of monitoring days is shown in brackets below the test label (i.e., T1 (20 d)). The days between the tests has also been included (i.e., the elapsed time between T1 and T2 was 58 days, 58 d).

			58 d				89 d				286 d				90 d			
	T1 (20 d)			T2 (13 d)				T3 (8 d)				T4 (8 d)				T5 (4 d)		
Parameter	Avg	SD		Avg		SD		Avg		SD		Avg		SD		Avg		SD
Walking hours	1.81	0.72		1.95		0.95		1.05		0.50		1.02		0.58		0.63		0.18
Sitting hours	9.15	1.55		10.76		0.96		9.17		0.95		11.38		2.42		8.30		0.43
Lying hours	1.01	0.69		0.87		0.49		1.40		0.74		1.69		1.29		1.53		1.37
Steps per day	5595	2158		6080		2672		3238		1586		2976		1699		1900		495
Speed (m/s)	0.98	0.09		0.98		0.06		0.81		0.17		0.76		0.08		0.90		0.04

**Table 4:** Statistical results of the frail (institutionalized) volunteer for each short-term test. Tx indicates the number of test (x = 1:5). The number of monitoring days is shown in brackets below the test label (i.e., T1 (11 d)). The days between the tests has also been included (i.e., the elapsed time between T1 and T2 was 94 days, 94 d).

			94 d				122 d				17 d				38 d			
	T1 (11 d)			T2 (26 d)				T3 (11 d)				T4 (8 d)				T5 (7 d)		
Parameter	Avg	SD		Avg		SD		Avg		SD		Avg		SD		Avg		SD
Walking hours	0.43	0.08		0.42		0.11		0.23		0.12		0.10		0.10		0.20		0.18
Sitting hours	9.47	0.58		9.44		0.62		9.38		0.53		9.03		0.76		9.64		0.77
Lying hours	1.60	0.39		1.62		0.37		1.87		0.41		1.88		0.80		1.78		0.45
Steps per day	1282	250		1175		549		800		348		56		50		629		465
Speed (m/s)	0.56	0.02		0.51		0.07		0.61		0.09		0.55		0.27		0.61		0.20
Floor changes per day	7.80	1.52		7.50		2.09		6.91		1.64		7.00		1.26		7.14		1.35

## Data Availability

Not applicable.

## References

[1] Rogers W.A., Mitzner T.L. (2017). Envisioning the future for older adults: Autonomy, health, well-being, and social connectedness with technology support. Futures.

[2] Binette J., Kerri V. (2018). 2018 Home and Community Preferences: A National Survey of Adults Age 18-Plus.

[3] Kim D., Bian H., Chang C.K., Dong L., Margrett J. (2022). In-Home Monitoring Technology for Aging in Place: Scoping Review. Interact. J. Med. Res..

[4] Olmedo-Aguirre J.O., Reyes-Campos J., Alor-Hernández G., Machorro-Cano I., Rodríguez-Mazahua L., Sánchez-Cervantes J.L. (2022). Remote Healthcare for Elderly People Using Wearables: A Review. Biosensors.

[5] Manickam P., Mariappan S.A., Murugesan S.M., Hansda S., Kaushik A., Shinde R., Thipperudraswamy S.P. (2022). Artificial Intelligence (AI) and Internet of Medical Things (IoMT) Assisted Biomedical Systems for Intelligent Healthcare. Biosensors.

[6] Izquierdo M., Duque G., Morley J.E. (2021). Physical activity guidelines for older people: Knowledge gaps and future directions. Lancet Healthy Longev..

[7] Langhammer B., Bergland A., Rydwik E. (2018). The Importance of Physical Activity Exercise among Older People. BioMed Res. Int..

[8] Rao A.K. (2019). Wearable Sensor Technology to Measure Physical Activity (PA) in the Elderly. Curr. Geriatr. Rep..

[9] Groessl E.J., Kaplan R.M., Rejeski W.J., Katula J.A., King A.C., Frierson G., Glynn N.W., Hsu F.-C., Walkup M., Pahor M. (2007). Health-related quality of life in older adults at risk for disability. Am. J. Prev. Med..

[10] Stessman J., Hammerman-Rozenberg R., Cohen A., Ein-Mor E., Jacobs J.M. (2009). Physical activity, function, and longevity among the very old. Arch. Int. Med..

[11] Yeom H.A., Fleury J., Keller C. (2008). Risk factors for mobility limitation in community-dwelling older adults: A social ecological perspective. Geriatr. Nurs..

[12] Hirvensalo M., Rantanen T., Heikkinen E. (2000). Mobility difficulties and physical activity as predictors of mortality and loss of independence in the community-living older population. J. Am. Geriatr. Soc..

[13] Li Y., Yan K. (2021). Indoor Location Based on Radio and Sensor Measurements. IEEE Sens. J..

[14] Bai L., Ciravegna F., Bond R., Mulvenna M. (2020). A Low Cost Indoor Positioning System Using Bluetooth Low Energy. IEEE Access.

[15] Minici D., Cola G., Giordano A., Antoci S., Girardi E., Di Bari M., Avvenuti M. (2021). Towards automated assessment of frailty status using a wrist-worn device. IEEE J. Biomed. Health Inform..

[16] Plaza S.L., Villadangos Carrizo J.M., García Domínguez J.J., Jiménez Martín A., Gómez D.G. FrailWear: A Wearable IoT Device for Daily Activity Monitoring of Elderly Patients. Proceedings of the XXXV Conference on Design of Circuits and Integrated Systems (DCIS) 2020.

[17] Shum L.C., Faieghi R., Borsook T., Faruk T., Kassam S., Nabavi H., Spasojevic S., Tung J., Khan S.S., Iaboni A. (2022). Indoor Location Data for Tracking Human Behaviours: A Scoping Review. Sensors.

[18] Alam F., Faulkner N., Parr B. (2021). Device-Free Location: A Review of Non-RF Techniques for Unobtrusive Indoor Positioning. IEEE Int. Things J..

[19] Yint Tun S.Y., Madanian S., Mirza F. (2021). Internet of things (IoT) applications for elderly care: A reflective review. Aging Clin. Exp. Res..

[20] Panhwar Y., Naghdy F., Naghdy G., Stirling D., Potter J. (2019). Assessment of frailty: A survey of quantitative and clinical methods. BMC Biomed. Eng..

[21] Roberts L.M., Jaeger B.C., Baptista L.C., Harper S.A., Gardner A.K., Jackson E.A., Pekmezi D., Sandesara B., Manini T.M., Anton S.T. (2019). Wearable Technology To Reduce Sedentary Behavior And CVD Risk In Older Adults: A Pilot Randomized Clinical Trial. Clin. Interv. Aging.

[22] Yuki A., Lee S., Kim H., Kozakai R., Ando F., Shimokata H. (2012). Relationship between physical activity and brain atrophy progression. Med. Sci. Sports Exerc..

[23] Rapp K., Mikolaizak S., Rothenbacher D., Denkinger M.D., Klenk J. (2018). Prospective analysis of time out-of-home and objectively measured walking duration during a week in a large cohort of older adults. Eur. Rev. Aging Phys. Act..

[24] Mahant P.R., Stacy M.A. (2001). Movement disorders and normal aging. Neurol. Clin..

[25] Ramezani R., Zhang W., Xie Z., Shen J., Elashoff D., Roberts P., Stanton A., Eslami M., Wenger N., Sarrafzadeh M. (2019). A Combination of Indoor Localization and Wearable Sensor–Based Physical Activity Recognition to Assess Older Patients Undergoing Subacute Rehabilitation: Baseline Study Results. JMIR mHealth uHealth.

[26] Fillekes M.P., Kim E.-K., Trumpf R., Zijlstra W., Giannouli E., Weibel R. (2019). Assessing Older Adults’ Daily Mobility: A Comparison of GPS-Derived and Self-Reported Mobility Indicators. Sensors.

[27] VandeWeerd C., Yalcin A., Aden-Buie G., Wang Y., Roberts M., Mahser N., Fnu C., Fabiano D. (2020). HomeSense: Design of an ambient home health and wellness monitoring platform for older adults. Health Technol..

[28] Hyväri S., Elo S., Kukkohovi S., Lotvonen S. (2022). Utilizing activity sensors to identify the behavioural activity patterns of elderly home care clients. Disabil. Rehabil. Assist. Technol..

[29] Forkan A.R.M., Branch P., Jayaramana P.P., Ferretto A. Halley Assist: A Personalised Internet of Things Technology to Assist the Elderly in Daily Living. Proceedings of the 52nd Hawaii International Conference on System Sciences.

[30] Alharbia M., Straitona N., Smithb S., Neubeckc L., Gallaghera R. (2019). Data management and wearables in older adults: A systematic review. Maturitas.

[31] Leirós-Rodríguez R., García-Soidán J.L., Romo-Pérez V. (2019). Analyzing the Use of Accelerometers as a Method of Early Diagnosis of Alterations in Balance in Elderly People: A Systematic Review. Sensors.

[32] Wipfli B., Rice S.P.M., Olson R., Ha K., Trullinger-Dwyer C., Bodner T. (2023). Describing Physical Activity Patterns of Truck Drivers Using Actigraphy. Saf. Health Work..

[33] Ummels D., Bijnens W., Aarts J., Meijer K., Beurskens A.J., Beekman E. (2020). The Validation of a Pocket Worn Activity Tracker for Step Count and Physical Behavior in Older Adults during Simulated Activities of Daily Living. Gerontol. Geriatr. Med..

[34] Chen Y.L., Yang I.J., Fu L.C., Lai J.S., Liang H.W., Lu L. (2021). IMU-Based Estimation of Lower Limb Motion Trajectory with Graph Convolution Network. IEEE Sens. J..

[35] Silsupadol P., Prupetkaew P., Kamnardsiri T., Lugade V. (2019). Smartphone-based assessment of gait during straight walking, turning, and walking speed modulation in laboratory and free-living environments. IEEE J. Biomed. Health Inform..

[36] Ramanujam E., Perumal T., Padmavathi S. (2021). Human Activity Recognition With Smartphone and Wearable Sensors Using Deep Learning Techniques: A Review. IEEE Sens. J..

[37] Joosen P., Piette D., Buekers J., Taelman J., Berckmans D., Boever P. (2019). A smartphone-based solution to monitor daily physical activity in a care home. J. Telemed. Telecare.

[38] NGIMU, X-IO. http://x-io.co.uk/ngimu/.

[39] Gualda D., Pérez-Rubio M.C., Ureña J., Pérez-Bachiller S., Villadangos J.M., Hernández Á., García J.J., Jiménez A. (2021). LOCATE-US: Indoor Positioning for Mobile Devices Using Encoded Ultrasonic Signals, Inertial Sensors and Graph-Matching. Sensors.

[40] Munoz–Diaz E., Mendiguchia–Gonzalez A.L. Step Detector and Step Length Estimator for an Inertial Pocket Navigation System. Proceedings of the International Conference of Indoor Positioning and Indoor Navigation (IPIN) 2014.

[41] TP-LINKTL-WR802N. https://www.tp-link.com/en/home-networking/wifi-router/tl-wr820n/#specifications.

[42] STMicroelectronics (2021). STM32F469xx Product Datasheet. https://www.st.com/resource/en/datasheet/stm32f469ae.pdf.

[43] CEVA (2023). BNO08X Data Sheet. https://www.ceva-dsp.com/wp-content/uploads/2019/10/BNO080_085-Datasheet.pdf.

[44] STMicroelectronics (2017). LPS22HB MEMS Nano Pressure Sensor: 260–1260 hPa Absolute Digital Output Barometer. https://www.st.com/resource/en/datasheet/lps22hb.pdf.

[45] Vishay Semiconductors (2003). TSOP7000. IR Receiver for High Data Rate PCM at 455 kHz. https://www.tme.eu/Document/0a7701c716b24d147be2f244f54bf755/tsop7000.pdf.

[46] Knowles (2012). SPU0414HR5H-SB Product Data Sheet. https://mm.digikey.com/Volume0/opasdata/d220001/medias/docus/1220/SPU0414HR5H-SB.pdf.

